# MeStanG—Resource for High-Throughput Sequencing Standard Data Sets Generation for Bioinformatic Methods Evaluation and Validation

**DOI:** 10.3390/biology14010069

**Published:** 2025-01-14

**Authors:** Daniel Ramos Lopez, Francisco J. Flores, Andres S. Espindola

**Affiliations:** 1 Institute for Biosecurity and Microbial Forensics (IBMF), Oklahoma State University, Stillwater, OK 74078, USA; aramosl@okstate.edu; 2 Department of Entomology and Plant Pathology, Oklahoma State University, Stillwater, OK 74078, USA; 3 Departamento de Ciencias de la Vida y la Agricultura, Universidad de las Fuerzas Armadas-ESPE, Sangolquí 171103, Ecuador; fjflores2@espe.edu.ec; 4 Centro de Investigación de Alimentos, CIAL, Facultad de Ciencias de la Ingeniería e Industrias, Universidad UTE, Quito 170527, Ecuador

**Keywords:** bioinformatics, metagenomics, high-throughput sequencing

## Abstract

Metagenomics analysis has enabled the measurement of the microbiome diversity in environmental samples without prior targeted enrichment. Functional and phylogenetic studies based on microbial diversity retrieved using HTS platforms have advanced from detecting known organisms and discovering unknown species to applications in disease diagnostics. Robust validation processes are essential for test reliability, requiring standard samples and databases deriving from real samples and in silico generated artificial controls. We propose a MeStanG as a resource for generating HTS Nanopore data sets to evaluate present and emerging bioinformatics pipelines. MeStanG allows samples to be designed with user-defined organism abundances expressed as number of reads, reference sequences, and predetermined or custom errors by sequencing profiles. The simulator pipeline was evaluated by analyzing its output mock metagenomic samples containing known read abundances using read mapping, genome assembly, and taxonomic classification on three scenarios: a bacterial community composed of nine different organisms, samples resembling pathogen-infected wheat plants, and a viral pathogen serial dilution sampling. The evaluation was able to report consistently the same organisms, and their read abundances as provided in the mock metagenomic sample design. Based on this performance and its novel capacity of generating exact number of reads, MeStanG can be used by scientists to develop mock metagenomic samples (artificial HTS data sets) to assess the diagnostic performance metrics of bioinformatic pipelines, allowing the user to choose predetermined or customized models for research and training.

## Simple Summary

Metagenomics analysis measures microbiome diversity in samples without prior enrichment. Advances in High-Throughput Sequencing (HTS) have expanded its use from identifying known organisms to diagnosing diseases. Reliable results need strong validation with standard samples and databases from real and synthetic controls. We introduce the Metagenomic Standards Generator (MeStanG), a tool for creating HTS Nanopore data sets to test bioinformatics pipelines. MeStanG allows users to design and generate samples with specific numbers of reads for each organism from reference sequences and error profiles. The accuracy was tested by simulating metagenomic samples with known diversities and abundances expressed as number of reads. The analysis showed results that matched the expected organism composition in the samples. MeStanG is a valuable tool for scientists to create mock metagenomic samples useful in diagnostic assay validation studies and assess bioinformatics pipeline performance using simulated samples.

## 1. Introduction

Advances in molecular biology and genomics made possible the assessment of the diversity, richness, and interaction of the organisms present in an environment sample [1]. Metagenome is a term used to refer to a collection of genomes in samples retrieved by amplicon and whole-genome shotgun strategies, focusing on microbial diversity and functional studies [2]. Metagenomics has been used to profile several ecosystems and environments’ taxonomic and functional interactions, making identifying specific microbes possible [3]. Direct sequencing of raw environmental DNA is used as a technique to retrieve quantitative sequence information from a sample, allowing the classification of known and the discovery of new taxa by association with known organisms [4,5]. Shotgun High-throughput sequencing (HTS) provides fast and extensive insights into massive biological data with different sequencing platforms including Illumina, Nanopore, PacBio, and Ion Torrent platforms, which are used for studies on phylogenetic, functional, and descriptive metagenomics [6,7,8]. Microbial diversity has been one of the leading research areas that have improved over the years since the advent of shotgun metagenomics. Several tools have been developed for reconstructing microbial composition [9], viral discovery [10], and unculturable organism detection [11]. One challenge posed by these techniques is the complex data analysis required to effectively establish shotgun metagenomics HTS as a strategy for pathogen detection and diagnostics regarding the massive amounts of data, diversity of highly specialized pipelines, and computationally expensive processes [12].

Validating the accuracy of read classification procedures can be challenging due to the lack of accurate reference databases, samples, and quality controls. To address this, in silico validation can be performed using sequence read simulators. When validating the accuracy of read classifiers, it is essential to consider the diversity of metagenomic samples, how accurately they resemble real samples, and determine their analytical sensitivity and specificity as a first validation tier [13,14]. Using sequence sets generated in silico representing the diverse naturally occurring sequencing outputs from real samples is crucial for validating diagnostic tests based on HTS methods [15].

There are several HTS simulators available for short (ART v.2.5.8, DWGSIM v.0.1.15, InSilicoSeq v.1.5.4, Mason v.2.0.9, NEAT v.3.0, wgsim v.0.3.1-r13) [16], long (NanoSim v3.1.0, HeteroGenesis v1.5, DeepSimulator v1.5) [17], and metagenomic reads (NanoSim v3.1.0, CAMISIM 1.3). CAMISIM can use ART, wgsim, and NanoSim for metagenomic simulation in its framework, being NanoSim the only one capable of generating Nanopore reads through error model characterization on sequencing outputs followed by read simulation or *de novo* direct simulation using reference genomes and pre-trained models obtained from mock data sets [18,19]. However, there are cases where the corresponding real sequencing outputs are not available for model characterization or the pre-trained models generate data sets with read abundance distributions unsuitable for estimating performance indicators in precise sample composition analyses, such as assessing the limits of Detection, Sensitivity, and Specificity, metrics required for diagnostic assay validation.

Here, we propose Metagenomic Standards Generator (MeStanG) as a resource for simulating *de novo* specific nanopore data sets resembling sequencing data. The simulated data can be used to evaluate existing and emerging bioinformatics pipelines designed to analyze HTS data for taxonomic classification and diagnostic purposes.

## 2. Materials and Methods

**Figure 1:**
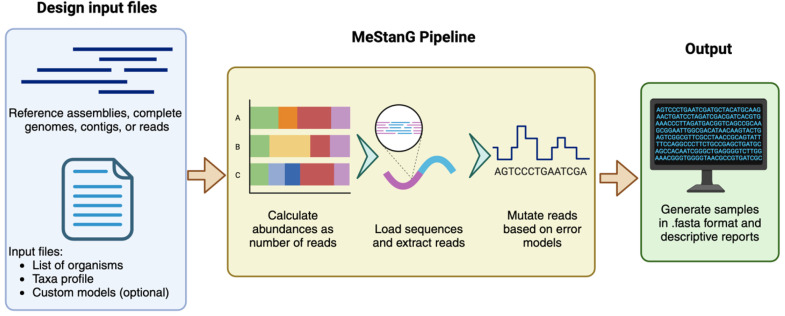
MeStanG workflow diagram. Created in BioRender [23].

MeStanG allows the generation of standard samples with precise composition features for the performance assessment of tools that rely on variable and abundances as number of reads per organism and diversities of the organisms in metagenomic communities. It is a *de novo* approach that requires reference data sets in FASTA format (assemblies, complete genomes, contigs, or reads) and a set of user-defined parameters for sample generation consistent with the reference sequence lengths. MeStanG introduces an algorithm for *de novo* error insertion resembling Guppy and Dorado base-calling performance implemented as error type [20,21,22] and specific base transition/transversion probability using an empiric model based on the chemical structures of the nucleotide nitrogenous bases. Users can also provide custom error rates and base-calling accuracy profiles (Figure 1).

Read abundance (RA) for the organisms in the metagenomic sample can be provided as (1) absolute number of reads, (2) relative to the total number of reads, or (3) assigned pseudo-randomly. Samples can be designed to resemble environmental or host-microbiome scenarios. Depending on the taxonomic distribution, the diversity design has two approaches: individual taxa or taxa with subtaxa. For designing diversity as individual taxa, each organism in the community exists as an independent taxon, and an assignment of absolute or relative RA is required individually. When designing diversity as taxa with subtaxa in scenarios where individual organisms’ RA cannot be provided or estimated but the information for a higher taxon is available, the RA for the higher taxon can be manually set and distributed among the subtaxa, i.e., a species complex with a total known RA but unknown for each organism in the group individually. Samples are generated in FASTA format, along with reports of the absolute and relative RA, error profile, error distribution, and run parameters.

To determine MeStanG’s capability of generating metagenomes with specified read length, depth, and taxon microbial RA when compared to NanoSim metagenome mode, several generated metagenomes with both simulation platforms using the same design parameters were evaluated using pipelines for metagenome analysis through assembly [24,25], mapping to reference [25], and taxonomic sequence classification [26].

### 2.1. Bacterial-Only Metagenome

Nine bacterial species assemblies stored in the National Center for Biotechnology Information (NCBI) database were used as input for generating a metagenome sample that resembles one that contains only bacterial organisms with MeStanG and NanoSim metagenome mode. *Bacillus subtilis* (GCF_000009045.1), *Escherichia coli* (GCF_000005845.2), *Enterococcus faecalis* (GCF_000393015.1), *Klebsiella pneumoniae* (GCF_000364385.3), *Limosilactobacillus fermentum* (GCF_029961225.1), *Listeria monocytogenes* (GCF_000438585.1), *Pseudomonas aeruginosa* (GCF_000006765.1), *Staphylococcus aureus* (GCF_000418345.1), and *Salmonella enterica* (GCF_000783815.2). MeStanG was run with parameters that aimed at generating an average read length of 2000 ± 200 nucleotides, varying the number of reads for each organism to get ~50× depth for assembly, optimal for the chosen read length [27].

Metagenome composition was detected through mapping to a combined reference of the nine bacteria using minimap2 v2.28-r1209 [28] and samtools v1.20 [29] for post-processing removing secondary and chimeric mappings, retrieving unique hits to each organism per read. Metagenome Assembly was performed with Miniasm v0.3-r179 [30] along with Racon v1.5.0 [31] for three polishing rounds and Flye v2.9.4-b1799 [32] optimized for metagenomic samples with three polishing rounds. Assemblies were evaluated using MetaQUAST v5.2.0 [33], and dot plots were generated using D-Genies for the assembly alignment to the combined reference [34]. Taxonomic sequence classification was carried out using Kraken2 v2.1.3 [35], followed by Bracken v2.9 [36], and displayed using Pavian v1.0 [37].

### 2.2. Host-Pathogen Metagenome Sample Generation

Fifteen metagenomic HTS data sets were generated with MeStanG and NanoSim, simulating bread wheat samples (Assembly accession: GCF_018294505.1) infected with three different pathogens. The pathogens included were *Puccinia striiformis* f. sp. *tritici* strain 134E16A+17+33+ (Assembly accession: GCF_021901695.1), *Xanthomonas translucens* pv. *undulosa* strain XtLr8 (Assembly accession: GCF_017301775.1), and Barley yellow dwarf virus—PAV (Nucleotide accession: NC_004750.1). Varying pathogen concentrations were used to resemble different host-pathogen interaction scenarios. Pathogens were detected on the samples using minimap2, Kraken2 followed by Bracken, and E-probe Diagnostic Nucleic Acid Analysis (EDNA) on Microbe Finder (MiFi^®^) [38,39]. Results were compared to the reported pathogen RA set in the sample design.

Additionally, a set of samples resembling five serial dilutions with 20 replicates each of wheat samples containing the viral pathogen Barley yellow dwarf virus were generated using MeStanG to evaluate the accuracy of RA design with the same approaches for detection previously described.

## 3. Results and Discussion

### 3.1. Bacterial-Only Metagenome Assessment

The results from mapping the bacterial metagenome to the combined reference genomes are consistent in mapping quality for MeStanG and NanoSim data sets, with higher accuracy in the number of mapped reads for MeStanG than NanoSim samples (Table 1).

**Figure 2:**
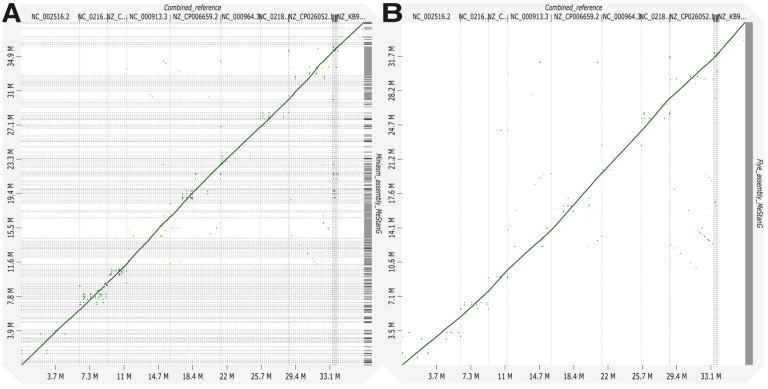
Genome alignment between the combined reference for nine different bacterial species to the metagenome assembly of the generated sample with MeStanG using (**A**) Miniasm + Racon and (**B**) Flye.

**Figure 3:**
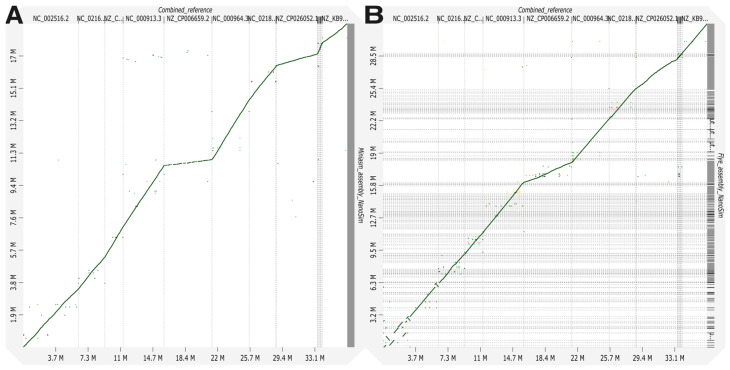
Genome alignment between the combined reference for nine different bacterial species to the metagenome assembly of the generated sample with NanoSim using (**A**) Miniasm + Racon and (**B**) Flye.

Assembly results for MeStanG generated samples with Miniasm followed by Racon polishing assembly genome fractions ranged from 95.890 to 99.392% for *S. enterica* and *B. subtilis*, respectively, and the dot plot displays a continuous high identity alignment to the combined reference genomes. Flye genome fractions ranged from 69.373 to 92.381% for *P. aeruginosa* and *E. faecalis*, respectively, the genome alignment plot has a similar disposition as the Miniasm assembly plot (Figure 2). Assembly results for NanoSim-generated samples with Miniasm followed by Racon polishing assembly genome fractions ranged from 37.440 to 99.621% for *K. pneumoniae* and *B. subtilis*, respectively, and the dot plot displays a discontinuous identity alignment for *K. pneumoniae* and *S. enterica*. Flye genome fractions ranged from 10.460 to 71.668% for *K. pneumoniae* and *L. fermentum*, respectively, the alignment plot has a similar disposition as the Miniasm assembly plot (Figure 3).

NanoSim simulation pipeline generates two sets of reads, aligned and unaligned. The latter contains reads simulated at random; merging the two sets gives the total number of reads specified for simulation [19]. Unaligned reads generation results in a ~13% loss in the RA initially specified by design calculated from all the results presented using mapping. This makes NanoSim unsuitable for approaches requiring an exact number of reads for analysis as it is not possible to modify how random reads are generated or estimate a proper initial relative RA to generate a specific number of reads.

Assembly metrics N50 refers to the contig length such that using contigs of the same size would produce half of the bases in the assembly, NGA50 is computed as the length of the aligned blocks that represent 50% of the reference genome size instead of the total assembly length [40]. While the N50 reflects the assembler performance in getting long contigs, NGA50 is a more informative metric for assembly completeness respect to a reference genome [41]. MetaQUAST fails to esteem NGA50 in four cases (Table 1) as the genome fraction is lower than 50%. The results suggest a better assembly performance for the MeStanG than the NanoSim sample based on NGA50 (the longer the better) and genome fraction metrics (the higher the better).

**Figure 4:**
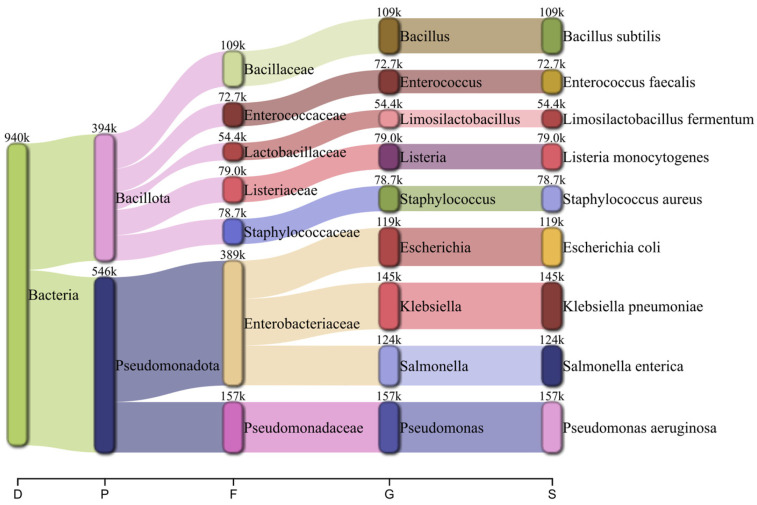
Pavian graph of the taxonomic diversity of the simulated metagenomic sample using MeStanG containing nine different bacterial species detected using Kraken2 followed by Bracken with the number of reads assigned to each organism. Taxonomic levels shown as D: Domain, P: Phylum, F: Family, G: Genus, S: Species.

**Figure 5:**
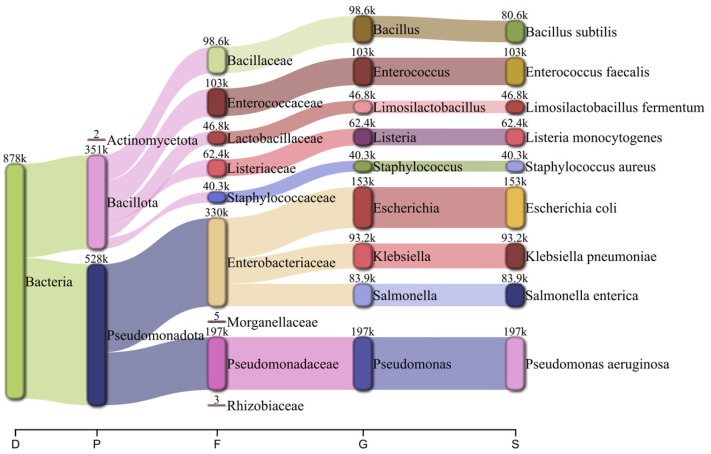
Pavian graph of the taxonomic diversity of the simulated metagenomic sample using NanoSim containing nine different bacterial species detected using Kraken2 followed by Bracken with the number of reads assigned to each organism. Taxonomic levels shown as D: Domain, P: Phylum, F: Family, G: Genus, S: Species.

Taxonomic classification using Kraken2 followed by Bracken reported RA consistent with the diversity distribution designed for the MeStanG sample, ranging from 96.735% for *K. pneumoniae* to 99.511% for *B. subtilis* (Figure 4). NanoSim sample reported RA ranged from 50.410% for *S. aureus* to 85.039% for *L. fermentum* with overestimations for *E. coli* (127.755%), *E. faecalis* (136.938%), and *P. aeruginosa* (123.163%) (Figure 5).

Kraken2 approach to classification is based on k-mers for efficient search against a database and it might misassign reads to a closely related species whose k-mers are similar within the same genus [42]. Bracken is used to improve the Kraken2 reported estimated RA at the species level leading to better estimates [43], however as Bracken redistributes RA at other taxonomic levels based on the initial classification, any unclassified reads with Kraken2 will likely remain unclassified. The classification accuracy for the bacterial-only sample generated with MeStanG was 98.417 ± 0.981%, consistent with the Bracken reported performance [36]. Accuracy for NanoSim generated samples was 69.320 ± 12.273% excluding the overestimations, this suboptimal read classification might be explained by the amount of unaligned random reads generated biased towards *E. coli* and *P. aeruginosa*.

### 3.2. Host-Pathogen Sample Generation

Results for mapping and EDNA-MiFi^®^ were consistent with the reported RA by MeStanG, with high-quality mappings for the pathogens present in the sample ranging from 16.928 to 60 (97.971 to 99.999% accuracy rate) and low-quality mapping scores when absent ranging from 0 to 5 (0 to 68.377% accuracy rate). NanoSim samples mappings were also consistent with high-quality mappings for the pathogens present ranging from 19.584 to 60 (98.899 to 99.999% accuracy rate) and low-quality mapping scores when absent ranging from 0 to 2.833 (0 to 47.917% accuracy rate) despite failing in retrieving all the intended abundancies for all samples as per previously discussed (Table 2).

Kraken2 classification was able to assign the RA consistently for MeStanG and NanoSim samples for each organism to the level of species as no specific strain/biotype was detected (PAV for Barley yellow dwarf virus/*Luteovirus pavhordei*, f. sp. *tritici* strain 134E16A+17+33+ for *Puccinia striiformis*, and pv. *undulosa* strain XtLr8 for *Xanthomonas translucens*) as Kraken2 might underestimate RA when classifying reads to the strain resolution level [42].

It is worth noting that taxonomic classification was more accurate in host-pathogen NanoSim samples compared to the results of the bacterial-only metagenome. This is likely due to the diversity in both cases being more homogeneous in the bacterial-only sample making it more difficult to discriminate between closely related organisms. On the other hand, taxonomic classification in MeStanG samples was consistent regardless of the diversity, making it suitable for generating samples to be subject of pipelines using high-accuracy analysis thresholds.

As a demonstration of the applicability of MeStanG in generating samples for assessing sensitivity of diagnostic tests, a total of 100 samples resembling a serial dilution routine were generated (Table 3)

The number of mapped reads and their mapping quality was the same throughout each of the 20 samples for all RA. Taxonomic classification was not able to estimate the exact number of reads for all samples, and EDNA-MiFi^®^ true positive rate decreases to 80% and 40% when there are 10 and five viral reads in the sample, respectively. The performance of taxonomic classification makes it more reliable than EDNA-MiFi^®^ detection in the lowest RA for this virus, which must be considered when using multiple pipelines for detection of pathogens in HTS samples.

The parameters used to generate the samples used in this study were set according to optimal values to ensure a proper metagenome assembly in terms of read length and number of reads, changes in the parameters will reflect in different sequencing depths obtaining better assemblies with higher depths [27,41]. Error rates from pre-trained models or customized models impact directly to the accuracy of the assembly and detection methods, making it necessary to have higher sequencing depths to address the unreliability generated by high error rates for assembly [22,41] and run polishing or correction pipelines to address possible misassemblies [44].

## 4. Conclusions

Based on its capacity of generating samples with exact number of reads per organism and the performance metrics evaluated using tools for detection of the read abundance and diversity of HTS samples, MeStanG has potential various applications, including creating standards for evaluating existing and emerging bioinformatics pipelines, generating controls for validation assays, improving the estimation of diagnostic tests sensitivity and specificity by generating exclusion and inclusion panels with sufficient replicates, benchmarking read classification systems based on sequence alignment by testing their performance on complex synthetic metagenome compositions resembling natural and artificial environments, and providing mock samples for teaching basic and advanced bioinformatic methods.

With the guidance of the user manual available at the MeStanG GitHub repository found in the Data Availability Statement section, we want to enable users to choose predefined reported performance models for common usage and customized profiles for research and training purposes depending on the requirements for sample generation, expecting to nurture more research based on artificial controls to estimate performance indicators before translating technologies into real scenarios.

## Figures and Tables

**Table 1:** Metagenome assessment statistics through mapping and assembly. SP: simulation platform; MeStanG (MSG) and NanoSim (NS), RA: Read abundances given as input for sample generation; absolute for MeStanG and relative for NanoSim, #reads mapped: number of best unique metagenome reads mapped to the respective reference organism genome, Mapq: mapping quality, MinR: assembly using Miniasm coupled with Racon, NA: data not reported by MetaQUAST.

Organism	SP	RA	# Reads Mapped	Mapq	Assembly Statistics with MetaQUAST							
					# Contigs		N50 (kbp)		NGA50 (kbp)		Genome Fraction (%)	
					MinR	Flye	MinR	Flye	MinR	Flye	MinR	Flye
*B. subtilis*	MSG	110000	110000	59.6	54	1850	436.656	2.302	436.656	2.303	99.392	83.513
	NS	11.518	98823	59.6	53	1335	906.356	2.389	897.144	2.274	99.621	68.313
*E. coli*	MSG	120000	119999	59.8	605	1741	29.770	2.325	34.623	2.311	96.015	75.711
	NS	12.565	109231	59.7	243	1369	56.722	2.407	61.704	2.269	98.073	64.021
*E. faecalis*	MSG	75000	74997	59.9	24	1414	322.042	2.180	321.587	2.194	97.577	92.381
	NS	7.853	64507	59.9	402	439	11.713	2.434	8.771	NA	94.618	35.953
*K. pneumoniae*	MSG	150000	150000	59.06	500	2764	59.197	2.253	61.164	2.259	96.337	88.924
	NS	15.707	143666	58.64	581	252	3.775	2.521	NA	NA	37.440	10.460
*L. fermentum*	MSG	55000	54998	59.7	81	999	88.468	2.186	86.192	2.192	97.247	89.566
	NS	5.759	45869	59.7	133	701	38.395	2.392	38.395	2.293	94.035	71.668
*L. monocytogenes*	MSG	80000	80000	59.65	89	1514	264.636	2.237	262.428	2.246	96.893	90.898
	NS	8.377	69162	59.65	93	743	79.871	2.431	59.473	2.240	95.488	54.202
*P. aeruginosa*	MSG	160000	160000	59.8	92	2091	304.150	2.353	304.150	2.325	97.888	69.373
	NS	16.754	165027	59.6	85	1260	296.041	2.424	242.860	NA	98.988	45.533
*S. aureus*	MSG	80000	80000	59.75	86	1116	136.220	2.340	136.220	2.325	97.494	75.045
	NS	8.377	69243	59.8	97	692	75.472	2.408	51.404	2.222	91.277	52.110
*S. enterica*	MSG	125000	125001	58.867	640	1793	31.082	2.337	35.386	2.321	95.890	74.873
	NS	13.089	113958	59.067	599	300	6.159	2.536	3.498	NA	57.236	15.050

**Table 2:** Host-pathogen sampling detection of select organisms using mapping, taxonomic classification, and EDNA-MiFi^®^. Sample: MSG—generated with MeStanG, NS—generated with NanoSim, RA: Read abundances given as input for sample generation; absolute for MeStanG and relative for NanoSim. # reads mapped: number of best unique metagenome reads mapped to the respective reference organism, Mapq: mapping quality, KB: reads assigned to each organism by Kraken2 + Bracken taxonomic classification, EM: EDNA-MiFi^®^ detection P for Positive and N for Negative.

Sample	Barley Yellow Dwarf Virus					*Puccinia striiformis* f. sp. *tritici*					*Xanthomonas translucens* pv. *undulosa*				
	RA	# Reads Mapped	Mapq	KB	EM	RA	# Reads Mapped	Mapq	KB	EM	RA	# Reads Mapped	Mapq	KB	EM
1_MSG	0	0	0	0	N	19233	19239	30.345	18433	P	0	5	0.333	0	N
1_NS	0	0	0	0	N	19.233	16337	31.923	16304	P	0	3	0.667	0	N
2_MSG	0	0	0	0	N	5303	5308	29.078	5081	P	0	2	5.000	0	N
2_NS	0	0	0	0	N	5.303	4283	29.400	4272	P	0	3	1.110	0	N
3_MSG	0	0	0	0	N	11571	11576	27.886	11377	P	9189	9196	59.967	9147	P
3_NS	0	0	0	0	N	11.571	9566	28.022	9551	P	9.189	7629	59.867	7611	P
4_MSG	0	0	0	0	N	7363	7373	21.974	6953	P	0	84	0.747	0	N
4_NS	0	0	0	0	N	7.363	6150	19.584	6141	P	0	159	1.087	0	N
5_MSG	19793	19793	60	18610	P	4084	4122	33.020	3933	P	0	19	1.157	0	N
5_NS	19.793	17337	60	17324	P	4.084	3346	32.354	3325	P	0	54	1.270	0	N
6_MSG	0	0	0	0	N	0	6	0.167	0	N	2758	2760	59.933	2654	P
6_NS	0	0	0	0	N	0	8	0.185	0	N	2.758	2102	59.000	2102	P
7_MSG	0	0	0	0	N	4476	4481	16.928	4266	P	5322	5322	59.933	5257	P
7_NS	0	0	0	0	N	4.476	3513	17.970	3503	P	5.322	4247	59.700	4244	P
8_MSG	3382	3382	60	3320	P	0	2	0.111	0	N	9310	9454	43.280	9260	P
8_NS	3.382	2700	60	2698	P	0	1	0.056	0	N	9.31	8096	42.063	7981	P
9_MSG	0	0	0	0	N	0	10	0.389	0	N	18520	18520	59.933	18099	P
9_NS	0	0	0	0	N	0	12	1.252	0	N	18.52	16025	59.500	16020	P
10_MSG	0	0	0	0	N	0	4	0.222	0	N	16140	16272	59.800	15824	P
10_NS	0	0	0	0	N	0	5	0.167	0	N	16.14	14013	59.533	13915	P
11_MSG	2947	2947	60	2831	P	658	663	28.289	642	P	0	2	1.500	0	N
11_NS	2.947	2324	60	2323	P	0.658	589	25.293	575	P	0	2	2.833	0	N
12_MSG	24813	24812	60	23676	P	0	4	0.389	0	N	0	8	2.500	0	N
12_NS	24.813	21568	60	21555	P	0	6	1.667	0	N	0	11	1.060	0	N
13_MSG	23626	23626	60	22534	P	0	6	0.019	0	N	0	2	0.333	0	N
13_NS	23.626	20117	60	20103	P	0	4	0.069	0	N	0	3	1.890	0	N
14_MSG	9560	9560	60	9280	P	0	13	0.619	0	N	0	0	0	0	N
14_NS	9.56	7666	60	7664	P	0	12	0.234	0	N	0	5	0.600	0	N
15_MSG	2123	2123	60	2039	P	0	10	0.454	0	N	3655	3656	59.967	3613	P
15_NS	2.123	1653	59.900	1654	P	0	3	1.500	0	N	3.655	2838	59.933	2838	P

**Table 3:** Simulated Serial dilution sampling with MeStanG of bread wheat plants infected with Barley yellow dwarf virus where each RA has 20 replicates displaying mean values with their corresponding standard deviation where available. Absolute RA: absolute read abundance used as input for number of reads simulation, Relative RA: relative read abundance respect to the total number of reads in the sample, # reads mapped: number of best unique reads mapped to the virus genome, Mapq: mapping quality, K2B: reads assigned to the virus by Kraken2 + Bracken taxonomic classification, EM (TPR%): EDNA-MiFi^®^ true positive rate detection.

Absolute RA	Relative RA (%)	# Reads Mapped	Mapq	Kraken2 + Bracken Hits	EM (TPR%)
500	0.5	500	60	490 ± 7	100
100	0.1	100	60	98 ± 1	100
50	0.05	50	60	48 ± 1	100
10	0.01	10	60	9 ± 1	80
5	0.005	5	60	4 ± 1	40

## Data Availability

The generated samples by MeStanG used to assess its performance can be found at https://doi.org/10.5281/zenodo.13858384. MeStanG source code and user manual can be found at: https://github.com/ibmf-bioinformatics/MeStanG, 11 December 2024.
